# Investigating Social Network Peer Effects on HIV Care Engagement Using a Fuzzy-Like Matching Approach: Cross-Sectional Secondary Analysis of the N2 Cohort Study

**DOI:** 10.2196/64497

**Published:** 2025-05-08

**Authors:** Cho-Hee Shrader, Dustin T Duncan, Redd Driver, Juan G Arroyo-Flores, Makella S Coudray, Raymond Moody, Yen-Tyng Chen, Britt Skaathun, Lindsay Young, Natascha del Vecchio, Kayo Fujimoto, Justin R Knox, Mariano Kanamori, John A Schneider

**Affiliations:** 1 Department of Epidemiology, Columbia University, 722 W 168th St, New York, NY, 10032, United States, 1 7033382642; 2 College of Nursing and Health Innovation, Arizona State University, Phoenix, AZ, United States; 3 New York Department of Mental Health and Hygiene, New York, NY, United States; 4 Fors Marsh, Arlington, VA, United States; 5 Population Health Sciences, University of Central Florida, Orlando, FL, United States; 6 University of Connecticut, Hartford, CT, United States; 7 Edward J. Bloustein School of Planning and Public Policy, Rutgers University, New Brunswick, NJ, United States; 8 School of Medicine, University of California San Diego, San Diego, CA, United States; 9 School for Communication and Journalism, University of Southern California, Los Angeles, CA, United States; 10 A Place for Rover, Seattle, WA, United States; 11 Center for Health Promotion and Prevention Research, School of Public Health, University of Texas Health Science Center at Houston, Houston, TX, United States; 12 Department of Psychiatry, Columbia University Irving Medical Center, Columbia University, New York, NY, United States; 13 New York State Psychiatric Institute, HIV Center for Clinical and Behavioral Studies, New York, NY, United States; 14 Department of Sociomedical Sciences, Columbia University, New York, NY, United States; 15 Department of Public Health Sciences, School of Medicine, University of Miami, Miami, FL, United States; 16 Department of Medicine, University of Chicago, Chicago, IL, United States; 17 Department of Public Health Sciences, University of Chicago, Chicago, IL, United States

**Keywords:** social network analysis, HIV, social media, social environment, sexual and gender minorities, African Americans, social network, social networking, public health, HIV care, health center, peer referral sampling, neighborhoods and networks, N2, Facebook, sexual health, mobile phone, fuzzy, innovation, digital health, mHealth, eHealth, surveillance, mobile health

## Abstract

**Background:**

Social network data are essential and informative for public health research and implementation as they provide details on individuals and their social context. For example, health information and behaviors, such as HIV-related prevention and care, may disseminate within a network or across society. By harmonizing egocentric and digital networks, researchers may construct a sociocentric-like “fuzzy” network based on a subgroup of the population.

**Objective:**

We aimed to generate a more complete sociocentric-like “fuzzy” network by harmonizing alternative sources of egocentric and digital network data to examine relationships between participants in the Neighborhoods and Networks (N2) cohort study. Further, we examined network peer effects of the status-neutral HIV care continuum cascade.

**Methods:**

Data were collected from January 2018 to December 2019 in Chicago, Illinois, United States, from a community health center and via peer referral sampling as part of the N2 cohort study, comprised of Black sexually minoritized men and gender expansive populations. Participants provided sociodemographics, social networks, sexual networks, mobile phone contacts, and Facebook friends list data. Lab-based information about the HIV care continuum cascade was also collected. We used an experimental approach to develop and test a fuzzy matching algorithm to construct a more complete network across social, sexual, phone, and Facebook networks using R and Excel. We calculated social network centrality measures for each of these networks and then described the HIV care continuum within the context of each network. We then used Spearman correlation and a network autocorrelation model to examine social network peer effects with HIV status and care engagement.

**Results:**

A total of 412 participants resulted in 2054 network connections (ties) across the confidant and sexual partner social networks (participants=387; ties=445), peer referral network (participants=412; ties=362), phone contacts (participants=273; ties=362), and Facebook network (participants=144; ties=1383), reaching the entire study sample in one fully connected “fuzzy” network. Results from the individual networks’ autocorrelation model suggest there are no peer effects on status-neutral HIV care engagement. Results from the final fuzzy-like sociocentric network autocorrelation model, adjusted for HIV serostatus, suggest that participants who were proximate to network members engaged in HIV care were significantly more likely to be engaged in care (ρ=0.128, SE 0.064; *P*=.045).

**Conclusions:**

Using alternative sources of network data allowed us to fuzzy match a more complete network: fuzzy matching may identify hidden ties among participants that were missed by examining alternative sources of network data separately. Although sociocentric studies require significant resources to implement, more complete sociocentric-like networks may be generated using a fuzzy match approach that leverages egocentric, peer referral, and digital networks. Enriching offline networks with digital network data may provide insights into characteristics and norms that egocentric approaches may not be able to capture.

## Introduction

Social epidemiology is the “branch of epidemiology concerned with the way that social structures, institutions, and relationships influence health.” [1] Within social epidemiology, social network models focus on how social networks can influence attitudes and knowledge surrounding health as they are a powerful force for influencing health behaviors [2,3]. As health information and behaviors can disseminate within a network, or across society [4], network science, a set of theories and methods for examining social relationships, has garnered interest among researchers, including social scientists and public health researchers, who are seeking to describe social phenomena [2,5-8]. Within network science, there is a fundamental dichotomy between sociocentric and egocentric network characterization [8]. Sociocentric study designs collect information about a “complete” network among members of a population or cohort of interest, whereas egocentric studies focus on the network of contacts that immediately surround a focal individual (ego) and often collect information from subgroups sampled from a larger population [1,9].

Sociocentric approaches are one of many frameworks for examining social network data; however, they are often perceived by researchers as the “gold standard” of network science in that they presumably include all nodes and ties within a network. The complexity and resource demands of collecting such data have led social scientists to use egocentric study designs, which are considered more pragmatic in that their elicitation can be more easily worked into traditional survey data collection. Despite their pragmatism, however, egocentric networks are limited as they intentionally ignore potential connections between ego networks and they tend to represent only a subsample of an existing network [5]. Given these tensions between the resource demands of sociocentric network data collection and the limits of egocentric network data collection, there is a need to find alternative sources of network data that could be collected from individuals without knowledge of who else is in the network and could be used to locate “hidden” connections among members of a focal population.

Individuals are often thought to have upwards of 1500 connections; crafting an egocentric survey design to capture all personal networks or a sociocentric approach to include all individuals bound within a network would be burdensome and analytically complex, although not impossible [10]. One way of bolstering traditional egocentric research and circumventing the steep demands of sociocentric designs is by incorporating digital epidemiology, which includes digital methods and data. Digital data includes mobile phone network data, data derived from the internet, and social media network data [11]. In the United States, cell phone and internet use are ubiquitous, with 97% and 95% of US adults owning a cell phone [12] and using the internet [13], respectively. Further, the majority of US adults frequent social networking websites such as YouTube (83%) and Facebook (68%) [14].

By harmonizing egocentric and digital networks, researchers may construct a “fuzzy” network based on a subgroup of the population. Although this more complete network would not be a “pure” sociocentric network as described by Laumann’s boundary specification [10,15], it could offer hidden ties that a purely egocentric network could not unveil. Deterministic linkage, a technique used to link records that may be less than a 100% match, could be used to triangulate egocentric data from multiple sources (eg, digital and phone networks) into a fuzzy network [16]. This matching compares all records of one dataset to another and creates matching pairs using prespecified linkage specifications such as phoenetic name and age, which are then manually validated to ensure a true match. Social network researchers could use deterministic linkage algorithms to explore if a study participant’s nominated tie (ie, alter) could be another participant in the study (ie, ego) [16]. Including additional types of networks such as mobile phonebook networks and online social networks could further aid the construction of a more complete network of a subgroup of a population with variable tie typologies that together may provide a more expansive understanding of social influence.

Using fuzzy matching and digital networks to generate connections across egocentric networks could provide insights into “hidden” relationships and social influence dynamics that are commonly excluded due to the limitations of egocentric approaches. Thus, our aim was to construct a fuzzy sociocentric-like network using egocentric confidant and sexual networks, peer referral ties, and digital networks (mobile phone and Facebook contacts), then to identify correlations between each of these networks and the fuzzy sociocentric-like network and status-neutral HIV care engagement among a cohort of Black sexually minoritized and gender expansive people (SGM) in Chicago [17]. This group is a relatively underreached group that continues to experience HIV-related health disparities at a disproportionate rate; approximately 1 in 3 Black sexually minoritized men are estimated to be diagnosed with HIV by age 50 if current rates persist [18,19]. As pre-exposure prophylaxis (PrEP) can reduce HIV acquisition by up to 99% [4-10], and antiretroviral treatment (ART) effectively eliminates forward transmission of HIV [11,12], together they can effectively end the HIV epidemic among Black SGM. However, Black SGM are not being reached by or using PrEP and ART at optimal rates to curb this HIV epidemic. Black SGM experience poor HIV-related outcomes in the United States due to historical and structural barriers and intersectional discrimination [20-23], which have manifested as reduced access to PrEP and ART. Although supportive social relationships are shown to promote PrEP and ART uptake [24], there is scant research that examines personal networks to identify the role of social networks on status-neutral HIV outcomes. A status-neutral approach proposes the same engagement approach for HIV prevention and care, regardless of HIV serostatus, and focuses instead on the common final state of engagement in HIV prevention (ie, PrEP) or care (ie, ART) activities [25].

Previous studies have suggested that social networks may be leveraged to surmount health disparities [26-31]; thus, this experimental approach is novel and important in reaching health equity for this marginalized group. Given that the data in the present study were collected 6 years ago, harmonization provides an opportunity to enhance their relevance and applicability by integrating them into a fuzzy-like sociocentric network. At the time that these data were collected, they were cutting edge, and despite 6 years passing since the collection of this data, HIV-related disparities in Chicago have exhibited stability over time, suggesting that the information derived from this dataset may still be applicable. To our knowledge, this robust dataset is the most updated and comprehensive social network dataset of Black SGM available since the uConnect cohort in Chicago [28], and it presents a unique opportunity for secondary analyses on a persistent health disparity (HIV) that disproportionately impacts Black SGM. For example, although HIV diagnoses in Chicago decreased by 7.7% from 2018 to 2022, Black non-Hispanic people (46.7% vs 47.1%) and men who have sex with men (60% vs 60.7%) continued to disproportionately comprise the same percentage of new HIV diagnoses while transgender women experienced an increase in new HIV diagnoses by 12.2%. This necessitates a better understanding of how networks may play a role in HIV prevention and care. Thus, the aim of this paper was to generate a more complete sociocentric-like “fuzzy” network by harmonizing alternative sources of egocentric and digital network data to examine relationships between participants in the Neighborhoods and Networks (N2) cohort study. Further, we aimed to examine the network peer effects of the status-neutral HIV care continuum cascade using our sociocentric-like “fuzzy” network.

## Methods

### Study Sample and Recruitment

Data were collected at baseline from January 2018 to December 2019 from the Chicago site of the multisite N2 cohort study, a prospective longitudinal study. Participants were recruited from a community health clinic and via peer referral in the South Side of Chicago, Illinois, United States. The community health clinic provides next-generation testing and notification, integrated biobehavioral HIV prevention, and community mobilization with the aim of eliminating new HIV transmission in the United States by 2041. The recruitment strategy was designed to leverage clients of the clinic and participants from existing research projects. We used a probabilistic variant of peer referral sampling that allowed participants to refer up to 6 members of their social networks. Participant inclusion criteria were the following: (1) Black or African American racial identity, (2) 16 years old or older, (3) assignment of male sex at birth, (4) residency in the Chicago Metropolitan Statistical Area (MSA), (5) having at least one sexual encounter with a cisgender man or transgender woman in the past year, (6) willingness to wear a GPS device, and (7) intention to remain in the Chicago MSA during the course of the study (~2 years). Additional information about the parent N2 study can be found elsewhere [32]. Given that the data in this study were collected 6 years ago, this analysis provides an opportunity to enhance their relevance by integrating them into a fuzzy-like sociocentric network. This paper was prepared using the Strengthening the Report of Observational Studies in Epidemiology (STROBE) guidelines [33].

### Data Collection

A study team member trained in quantitative and network data collection obtained written informed consent from participants and then collected data using interviewer-administered computer-assisted assessments in a private room at the study site. We collected the following data from each participant: sociodemographics, confidant and sexual partner information, mobile phone contact lists, and Facebook friends lists.

Sociodemographic data about the participant and each confidant and sexual partner included first name, last name, gender identity (cisgender man, cisgender woman, transgender or another gender expansive identity), race (Black or not Black), Latine ethnicity (Latine, not Latine), and age (in years). Sociodemographic data were collected using a network inventory using Qualtrics. We downloaded participants’ phone contact information and Facebook network information. We attempted the download of phone contacts and Facebook friends one time, and if the download was not successful then we considered that participants’ data to be missing at random.

### Analysis

#### Overview

We developed and tested a fuzzy matching algorithm (ie, deterministic linking algorithm) with a set of identifiers that provided a match result across confidant, sexual, phone contact, and Facebook networks. We used R (version 12.1.402; R Foundation for Statistical Computing [34]) and Microsoft Excel (Microsoft Corp) for data analysis and matching. The boundaries of the final complete sociocentric-like fuzzy network were only those participants enrolled in the N2 cohort study and did not include those individuals that participants may have named as being in their social networks. We describe the following characteristics for each of the networks: connected components (number of unique networks within the larger network), network density (proportion of nodes that are connected relative to all potential connections), degree (average number of nodes that a node reports knowing added to average number of nodes who also know the node), diameter (maximum distance between the furthest pair of nodes), radius (maximum shortest distance between pairs of nodes), betweenness centrality (how often a node acts as a bridge along the shortest path between two nodes), and characteristic path length (average shortest distance for all possible pairs of network nodes). We conducted an ANOVA to assess the differences in mean betweenness centrality based on HIV care cascade outcome (ie, not using PrEP, using PrEP, not virally suppressed, virally suppressed).

#### Matching Confidants With Sexual Partners

To identify confidants who were also sexual partners, we collected these data separately. Thus, participants named up to 5 people they considered to be their closest social network members using the prompt, “Who is the person you most talk to about things that are important to you?*”* Participants also named up to 5 people with whom they had sex with in the past 6 months. Participants indicated whether they had sex with a confidant and if a sexual partner was also a confidant. Egocentric network members included those who were confidants or sexual partners.

#### Matching Social Network Members With Participants Enrolled in N2

We adapted a previously used deterministic linkage algorithm [16]. We first phoneticized first and last names to standardize names in the instance of incorrect or multiple spellings of names. Thus, each name was given a unique phonetic code. We then used a *for loop* in R to match each social network member with each participant (dyad) based on the following selection criteria: phoneticized first name (both individuals have the same phoneticized first name), phoneticized last name (both individuals have the same phoneticized last name), gender identity (both individuals are homophilous on gender), race (both individuals within the pair being matched are homophilous on race), Latine ethnicity (both individuals within the pair being matched are homophilous on Latine ethnicity), and age (both individuals within the pair being matched are within 5 years of each other, age_i_ ± 5 years to age_j_).

Following a similar algorithm as in previous work, we created an autoscoring algorithm ranking the certainty of each dyad being compared [35]. We then manually reviewed each potential match of dyads to identify whether social network members named in the inventory could be matched to participants enrolled in the study. We decided that if dyads fit the following criteria, they were the same person: both members of the potential dyadic match (1) had the same phoneticized first name, phoneticized last name, or phoneticized first and last name; (2) were either both cisgender women, were both cisgender men, were both transgender-identified, or one member of the potential match dyad was transgender-identified and another member of the potential match dyad was a cisgender man; (3) were both Black or were both not Black; (4) and were within 5 years of age. We decided to match individuals who were indicated as being transgender with those who were indicated as being a cisgender man because we recognized that not all individuals may be “out” with their social networks and because gender can be fluid, and great strides in gender identity can be made in a short time. In addition, we considered a match on Latine identity to contribute toward the manual match algorithm; however, if both members of a dyad were not Latine, this did not negate the potential match.

#### Identifying Ties Between Participants Using Peer Referral Ties

We identified ties between participants using the peer referral ties during enrollment. Thus, we could see which participants were referred by an existing participant in the study.

#### Matching Participants Based on Mobile Phone Contact Data

We identified matches between participants’ phone contact data and other participants in the study based on the phone number provided at baseline data collection.

#### Matching Participants Based on Facebook Friends Lists

Facebook data were cleaned using Python (including the packages *beautifulsoup4.9.3*, *soupsieve2.2.1*, and *pandas1.2.4*) to parse through the files each participant’s information was stored in. The only available information for a Facebook friend was their display name—there was no additional identifying information for friends lists such as an identifier or URL. For this reason, we assumed any common Facebook friend was one held in common between participants, rather than assuming that 2 different people with the same name had friendships among the study population.

#### Network Peer Effects on HIV Engagement in Care Analysis

We investigated if there was an association between individuals’ HIV-related care and their network members’ engagement in status-neutral HIV-related care using a multistep process. We first identified the participant’s HIV serostatus (ie, 0=not living with HIV; 1=living with HIV) and their network members’ mean serostatus (ie, proportion of members living with HIV). We then identified the participant’s engagement in status-neutral HIV-related care (ie, 0=not engaged in care or not using ART/PrEP; 1=engaged in care or virally suppressed or using PrEP) and their network members’ mean engagement. For both of these outcomes (ie, HIV serostatus and engagement in status-neutral HIV-related care), we conducted the following steps:

Step 1: Spearman correlation to examine binary correlations between an individual’s outcome and the mean of their network’s outcome.
Step 2: Unadjusted linear regression to examine associations between an individual’s outcome and the mean of their network’s outcome.
Step 3: Quadratic Assignment Procedure (QAP) correlation to examine the relationship between an individual’s outcome and the mean of their network’s outcome. This allowed for the identification of the product-moment correlation between the adjacency matrices of graphs for an individual’s outcome and the mean of their network’s outcome [36]. We used the R environment packages *igraph* [37] and *statnet* [38] to use the *gcor*() function for analysis.
Step 4a: Linear Network Autocorrelation Model (LNAM) to examine the relationship between an individual’s outcome and the mean of their network’s outcome [39-41]. This model “assumes that social influence occurs when individuals are exposed to a behavior by their network contacts.” [42] We used the R environment package *sna* and the *lnam*() function [43].
Step 4b: LNAM to identify the social cross-sectional peer effects of a participant’s engagement in status-neutral HIV-related care within each of the networks, adjusting for their social network members’ HIV serostatus. We used the R environment package *sna* and the *lnam*() function [43].

The network autocorrelation model assumes continuous dependent variables; however, our outcome variables are binary. Because there is no established statistical model that can account for the network autocorrelation for the Bernoulli distribution of the error term, we used the LNAM. This model assumes a normal distribution of error terms with a null value and variance in the identity matrix. Due to this limitation, we added the supplemental analysis of conducting logistic regression as we could not adjust for the limitation of not accounting for potential network autocorrelation. Within the network autocorrelation model [39,44-46], we specified the network effects model, that is defined in the following formula:


$$y=\rho Wy+X\beta +\varepsilon ,\;\varepsilon ∼N\left(0,\sigma^{2}I\right)$$

in which y is a vector for values of our outcome variable (ie, engagement in status-neutral HIV-related care), and X is a matrix of values for the N actors. The **β** term is a vector of regression coefficients, ρ is a scalar estimate of the autocorrelation parameter, and W is a matrix of weights, which is the allocation of peer effects among network members (eg, engagement in HIV-related care). We also fit the multiple network autocorrelation model [40],

We also fit the multiple network autocorrelation model [40]

which was adjusted to include HIV serostatus (Xβ), as our unadjusted model does not include HIV serostatus Xβ.

### Network Visualization

We used Cytoscape to visualize participants’ peer referral ties; sexual, social, phone contact, and Facebook networks; and the final fuzzy match networks [47].

### Ethical Considerations

The N2 cohort study protocol for the Chicago site was approved by the Biological Sciences Division/University of Chicago Medical Center Institutional Review Board (IRB16-1419) and Columbia University (AAAS7654). Written informed consent was obtained from the study participants. Participants were compensated US $150 for their time and US $20 for each person they referred to the study. A Certificate of Confidentiality from the National Institute of Health was obtained to protect study participants’ privacy.

## Results

### Overview

A total of 412 Black SGM enrolled in the N2 cohort study. Of participants, 387 provided confidant and sexual network data, 273 provided mobile phone contact data, and 144 provided Facebook data. Of these networks, the Facebook network had the highest number of undirected ties (n=1383; undirected means that if one person in the network reported knowing the other, then they were considered to know each other, regardless of the direction of the tie), the fewest connected components, the highest density (13.4% of all potential ties), highest degree (n=19.21), and lowest network diameter (n=5), network radius (n=3), and average characteristic path length (length=2.28). The fuzzy match network consisted of 400 unique nodes and 12 isolates. There was a total of 2054 undirected edges in one centralized component after removing isolates. Its density was 0.026, with an average degree of 10.27. The network diameter was 9, the radius was 5, and the characteristic path length was 3.49. An overview of each of the networks, including nodes, undirected ties, and other network characteristics, can be found in Table 1.

**Table 1.** Overview of social network characteristics among 412 Chicago-based Black sexually minoritized and gender expansive people in the Neighborhoods and Networks (N2) cohort study (2018‐2019), with isolates removed, by network type.

	Confidant and sexual network	Peer referral network	Phone contact only	Facebook only	Entire sociocentric network (fuzzy)
Participants who provided data, n	387	412	273	144	412
Egos in network, n	379	344	245	144	400
Undirected ties within network, n	445	362	362	1383	2054
Connected components, n	10	19	12	1	1
Network density, mean	0.007	0.012	0.015	0.134	0.026
Network degree, mean	2.42	1.99	3.18	19.21	10.27
Network diameter, n	19	18	12	5	9
Network radius, n	11	9	7	3	5
Betweenness centrality, mean	0.08	0.03	0.03	0.009	0.006
Characteristic path length, mean	7.77	7.64	4.93	2.28	3.49
Number of HIV-negative people (%)	203 (52.5)	189 (45.8)	163 (59.7)	79 (54.9)	224 (54.4)
Number of people engaged in care (%)	200 (51.7)	194 (47.1)	144 52.7)	62 43.1)	179 (43.4)

### Matching Social Confidants With Sexual Partners

Of 412 participants, 387 completed the social network inventory. Participants named up to 5 social network alters (n=1036; mean 2.73, SD 1.32) and 5 sexual partner alters (n=1017; mean 2.57, SD 1.58). We identified an overlap of 136 confidants who were also sexual partners, for a total of 1917 unique alters.

### Matching Social Network Members With Participants Enrolled in N2

**Figure 1.**
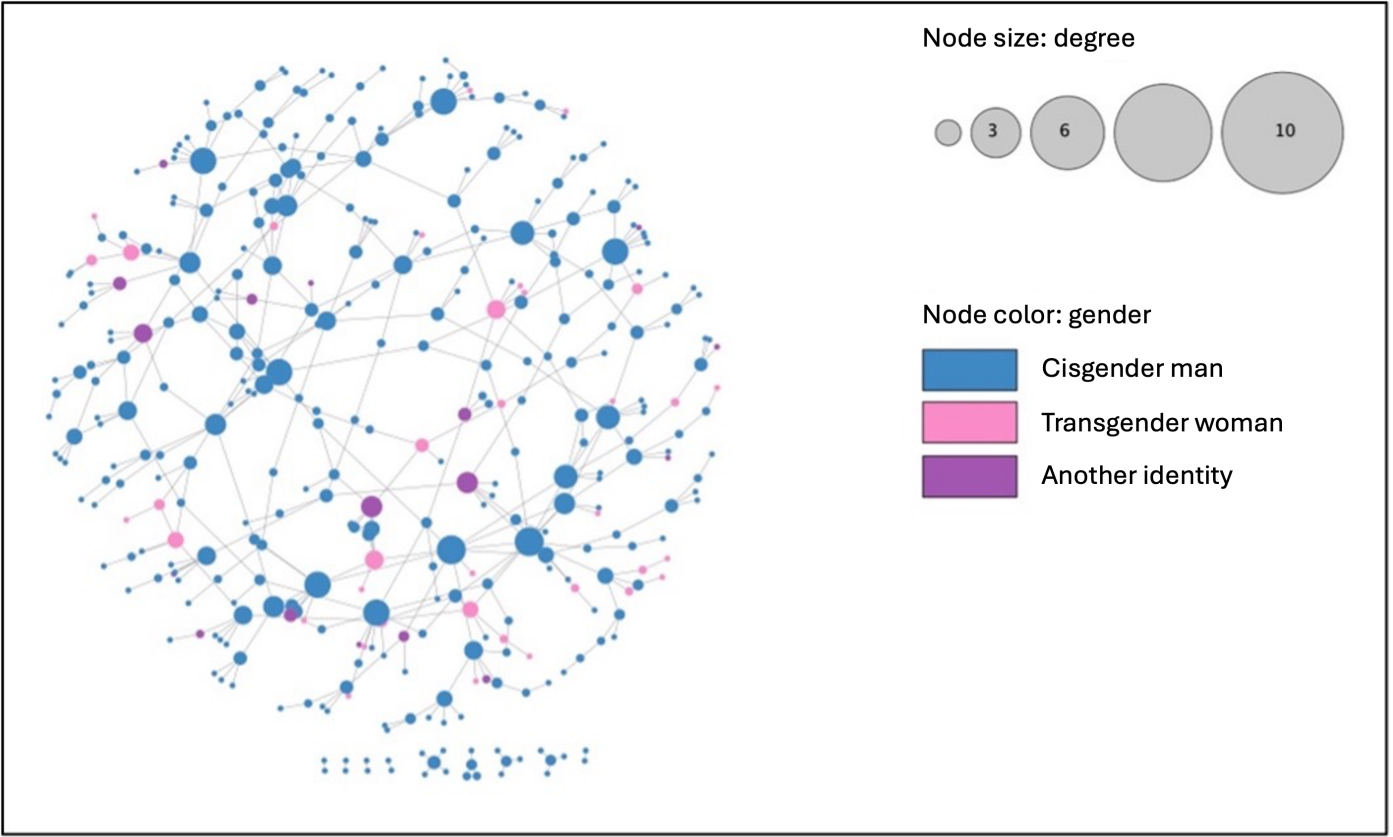
Egocentric confidant and sexual partner network of Chicago Black sexually minoritized and gender expansive people from the Neighborhoods and Networks (N2) cohort study (n=379), edge-weighted spring embedded layout by edge-betweenness.

There were a total of 1917 unique alters. When considering that 412 participants were enrolled in the N2 study, this means that there was a potential of 169,332 directional matches our analyses could identify (ie, A knowing B is treated as separate from B knowing A; between 412 participants, there are 84,666 potentially unique ties as 412 × 411 = 169,332). We found that there were 8020 potential “fuzzy” matches. Upon manual validation, we found that there were 445 undirected ties between the 379 nodes in this network. There were 10 connected components, and the network density was 0.007, indicating it was a sparse network. Average degree was 2.42 and betweenness centrality was 0.028. The network diameter was 19 steps, the radius was 11 steps, and the characteristic path length was 7.77 steps. The egocentric network can be observed in Figure 1. There was no statistical association between betweenness centrality and the HIV care cascade (*F*_3_=0.242; *P*=.87).

### Identifying Ties Between Participants Using the Peer Referral Sampling Chain

**Figure 2.**
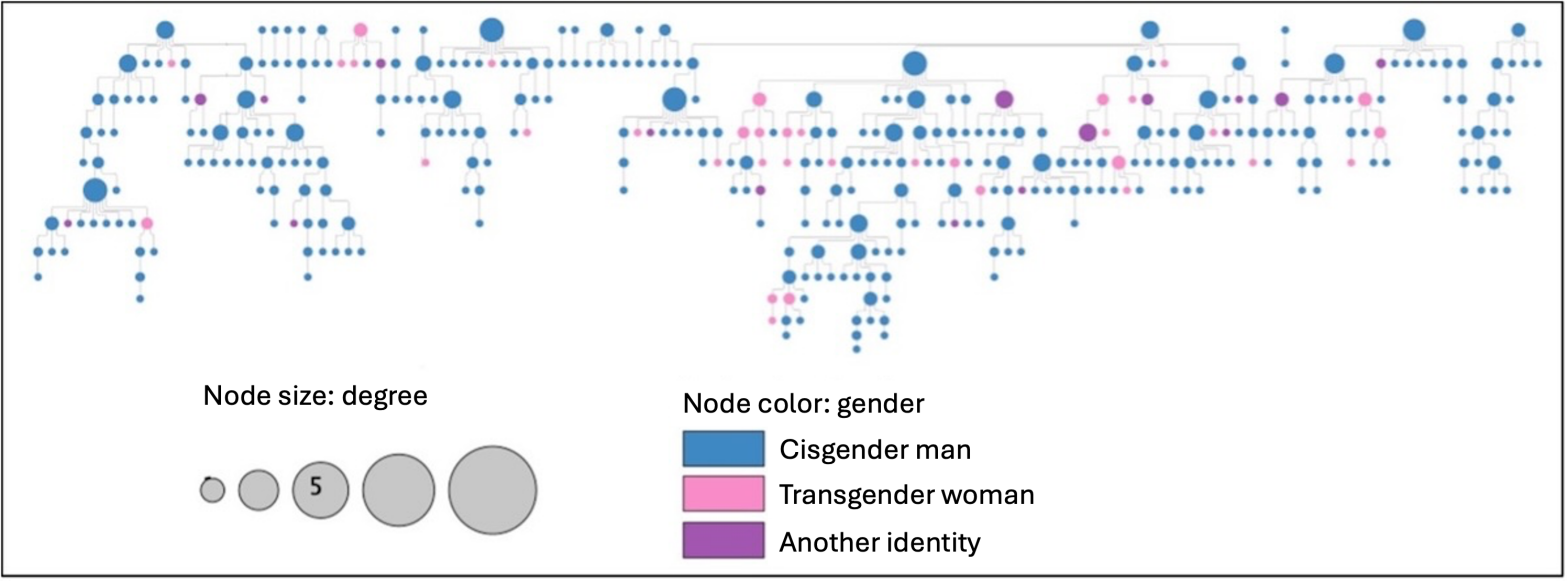
Peer referral tie network of Chicago Black sexually minoritized and gender expansive people from the Neighborhoods and Networks (N2) cohort study (n=344), yFiles hierarchic layout.

We identified 363 unique ties between 344 participants from the peer referral ties. There were 19 connected components (ie, 19 seeds), and the network density was 0.012. The average degree was 1.99 and betweenness centrality was 0.080. The network diameter was 18 steps, the radius was 9 steps, and the characteristic path length was 7.64 steps. The peer referral tie network is visualized in Figure 2 using a *yFiles* hierarchic layout to display the chain [48]. There was no statistical association between betweenness centrality and the HIV care cascade (*F*_3_=2.00; *P*=.11).

### Matching Participants Based on Mobile Phone Contact Data

**Figure 3.**
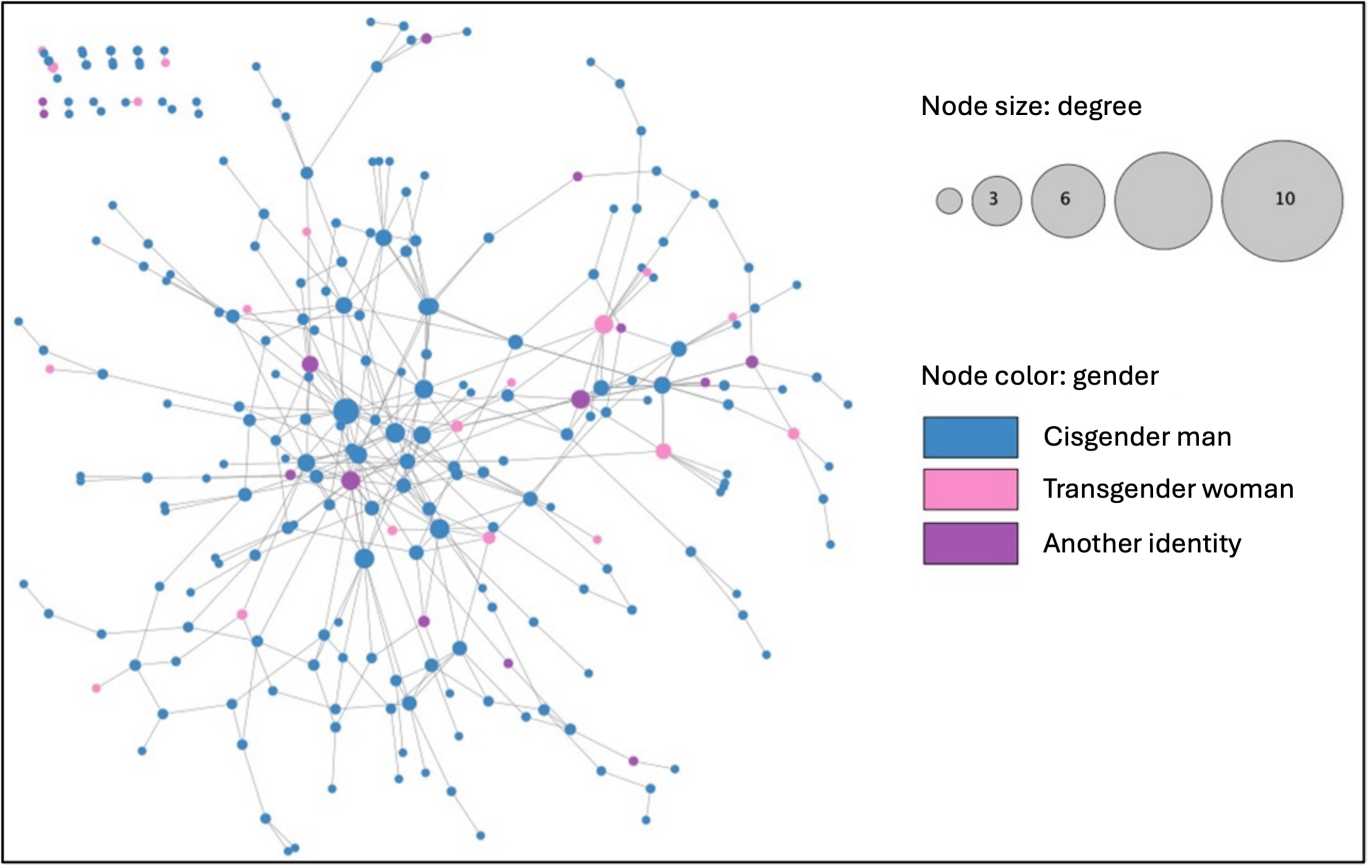
Phone contact network of Chicago Black sexually minoritized and gender expansive people from the Neighborhoods and Networks (N2) cohort study (n=245), edge-weighted spring embedded layout by edge-betweenness.

Of the participants, 294 provided consent to share their mobile phone contact data. There were 95,831 phone number entries, of which 51,671 were valid phone numbers (ie, in a 10-digit format). We found that there were 416 unique ties among 270 participants from the mobile phone contact data. Figure 3 displays a visualization of the mobile phone contact network. The average degree was 3.18 and betweenness centrality was 0.03. There was no statistical association between betweenness centrality and the HIV care cascade *(F*_3_=1.03; *P*=.39).

### Matching Participants Based on Facebook Data

**Figure 4.**
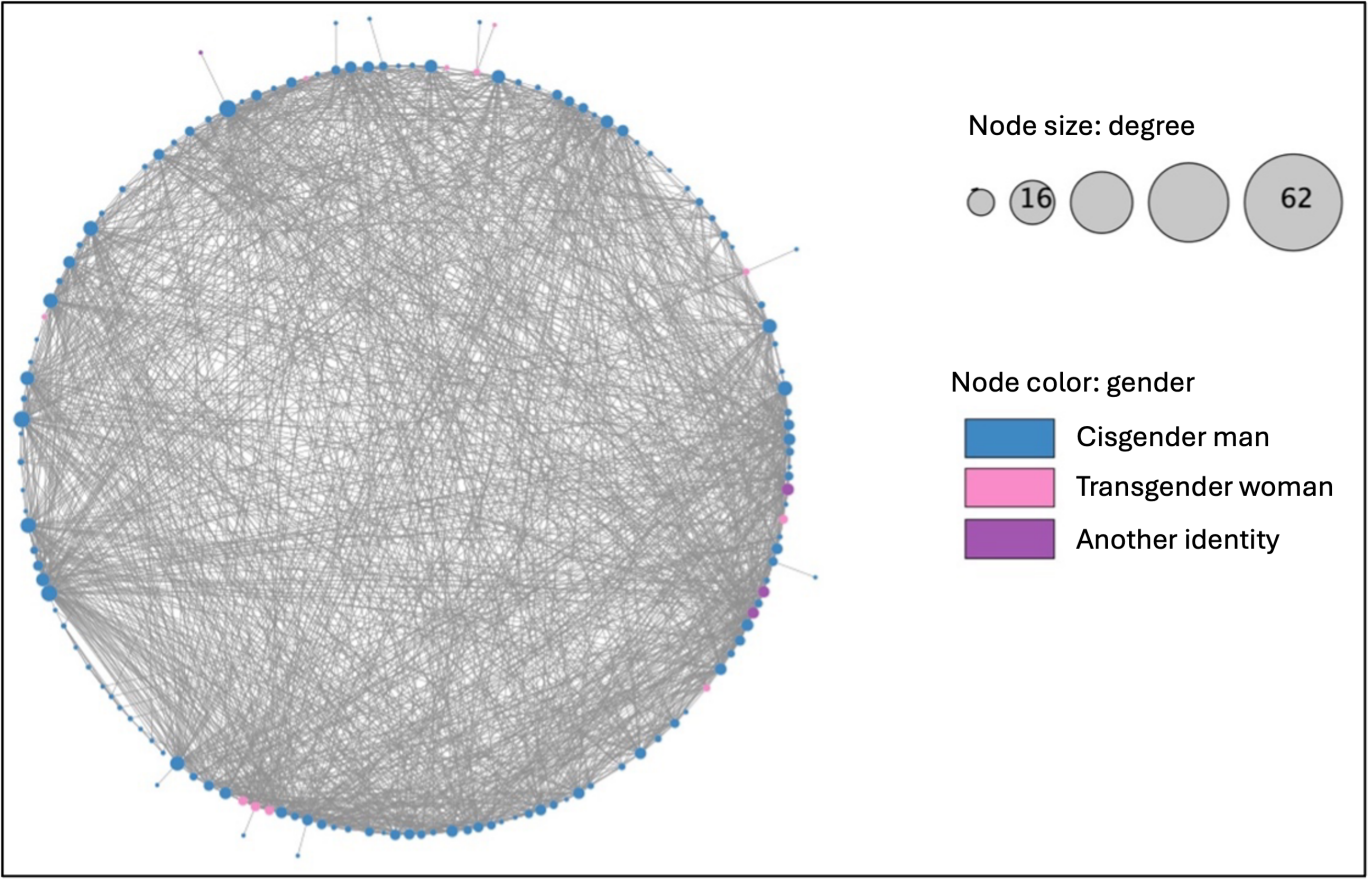
Facebook network of Chicago Black sexually minoritized and gender expansive people from the Neighborhoods and Networks (N2) cohort study (n=144), circular layout.

We downloaded the Facebook friends list of 144 participants. Due to refusal and interruptions in the download process, we were not able to download data for 268 participants. There were a total of 18,255 Facebook friendship ties, posts, and comments. Among these, we identified 1383 undirected ties between N2 participants. There was one connected component with a density of 0.134. The average degree was 19.21 and betweenness centrality was 0.009. The network diameter was 5 steps, the radius was 3 steps, and the characteristics path length was 2.28 steps. The Facebook-only network is visualized in Figure 4. There was no statistical association between betweenness centrality and the HIV care cascade (*F*_3_=1.194; *P*=.31).

### Fuzzy Match Network Description

**Figure 5.**
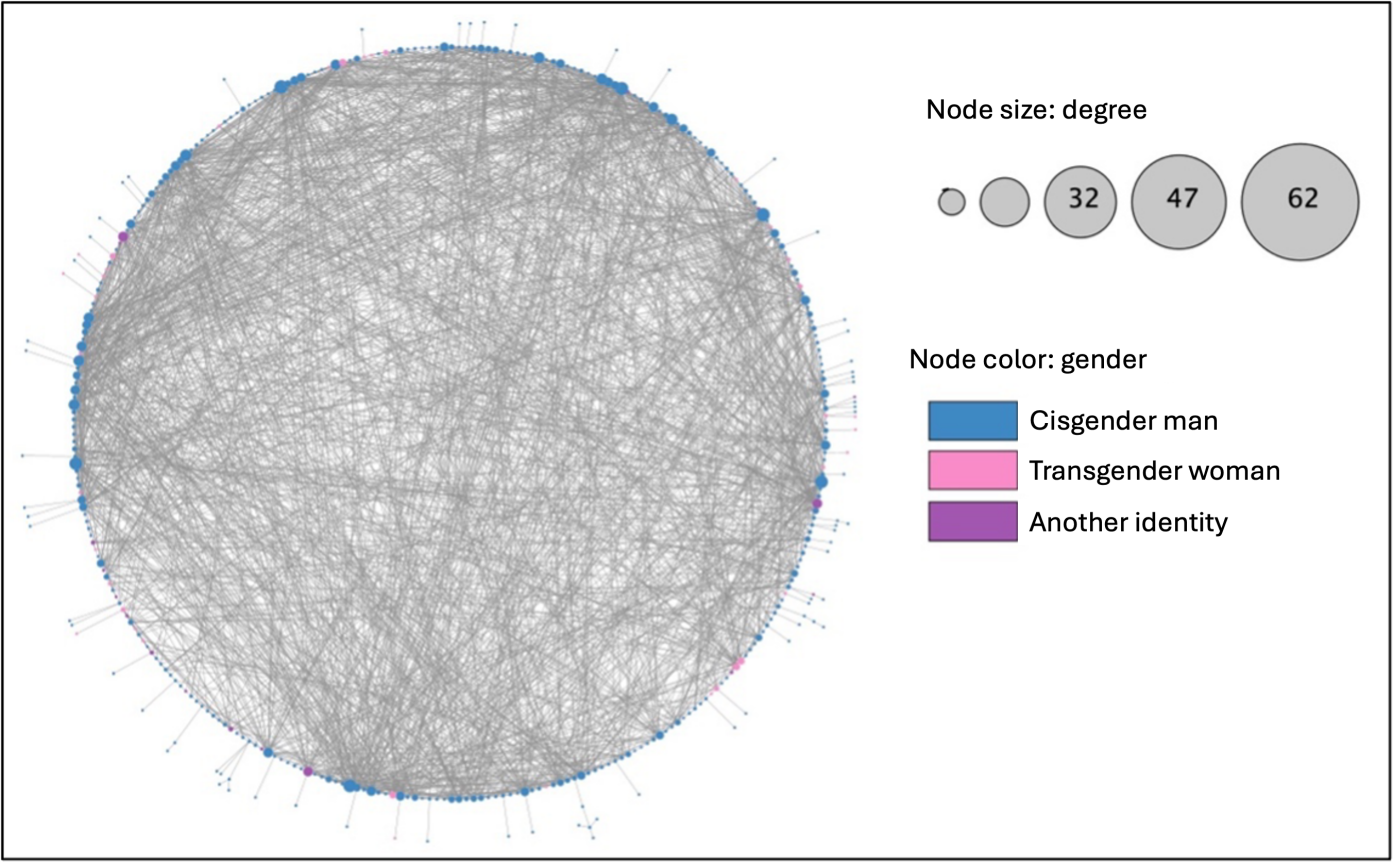
Sociocentric fuzzy match network of Chicago Black sexually minoritized and gender expansive people from the Neighborhoods and Networks (N2) cohort study (n=400), circular layout.

Once all networks were combined, we identified 400 participants who were included in the fuzzy-like sociocentric network. There were 2054 unique ties among participants. The density of our fuzzy match network was 0.025, and there was one connected component. The average degree was 10.21 and betweenness centrality was 0.006. The network diameter was 9 steps, the radius was 5 steps, and the characteristic path length was 3.49 steps. The fuzzy match sociocentric network is visualized in Figure 5. There was a statistically significant difference between betweenness centrality and the HIV care cascade (*F*_3_=5.837; *P*<.001). We conducted a post hoc comparison using the Tukey Honestly Significant Difference test and found that the not virally suppressed group had higher betweenness centrality measures relative to the group not using PrEP (Diff=0.006, 95% CI 0.002 to 0.009; *P*<.05); the PrEP-using group had lower betweenness centrality measures relative to the not virally suppressed group (Diff=−0.005, 95% CI −0.009 to −0.007; *P*<.05); and the virally suppressed group had lower betweenness centrality relative to the not virally suppressed group (Diff=−0.005, 95% CI −0.009 to –0.001; *P*<.01). The other 3 conditions were not statistically significant (*P*>.05).

### Findings of the Correlation, Quadratic Assignment Procedure Correlation, Regression Models, and Network Autocorrelation Models

We found a low correlation and QAP correlation between HIV status and care engagement between participants and their network members (Table 2). Only the regression model for engagement in care was found to be statistically significant; participants had a 2.07 higher odds ratio (95% CI 1.09‐3.97; *P*=.03) of engagement in care with higher network-level engagement in care. None of the unadjusted linear network autocorrelation models were significant when examining HIV status and care engagement for each of the different networks (Table 2, models a-e). For the linear network autocorrelation models adjusted for HIV status (Table 2, models f-j), HIV status had a significant negative association with care engagement for all network models: confidant and sexual (*β*=−0.268, SE 0.048; *P*<.001), peer referral (*β*=−0.281, SE 0.049; *P*<.001), phone contact–only (*β*=−0.231, SE 0.056; *P*<.001), Facebook-only (*β*=−0.252, SE 0.079; *P*=.001), and the combined sociocentric network (*β*=−0.256, SE 0.047; *P*<.001). Results from the linear network autocorrelation model for the combined sociocentric network adjusted for HIV status showed that care engagement had network clustering. Thus, participants who were proximate to network members who were engaged in care were significantly more likely to be engaged in care (ρ=0.128, SE 0.064; *P*=.045).

**Table 2.** Results of the unadjusted and adjusted network autocorrelation assessing the peer effects of status-neutral HIV outcomes among 412 Chicago-based Black sexually minoritized and gender expansive people in the Neighborhoods and Networks (N2) cohort study (2018‐2019), by network type.

	Confidant and sexual network	Peer referral network	Phone contact only	Facebook only	Entire sociocentric network (fuzzy)
**Spearman correlation between participant and average score of their network members**					
HIV serostatus	0.054	0.018	0.021	−0.119	0.004
Engagement in care	0.014	0.043	0.068	0.149	0.114
**Logistic regression analyses between participant and average score of their network members**					
HIV serostatus	OR 1.30, 95% CI 0.68‐2.46; *P*=.43	OR 1.08, 95% CI 0.70‐1.64; *P*=.74	OR 1.02, 95% CI 0.52‐2.38; *P*=.77	OR 0.38, 95% CI 0.09‐1.46; *P*=.17	OR 1.02, 95% CI 0.54‐1.93; *P*=.94
Engagement in care	OR 1.07, 95% CI 0.55‐2.10; *P*=.84	OR 1.20, 95% CI 0.77‐1.88; *P*=.44	OR 1.41, 95% CI 0.68‐2.92; *P*=.36	OR 3.51, 95% CI 0.86‐15.53; *P*=.09	OR 2.07, 95% CI 1.09‐3.97; *P*=.03^a^
**Quadratic Assignment Procedure correlation between participant and difference in the average score of their network members**					
HIV serostatus	−0.003	−0.003	−0.004	−0.007	−0.004
Engagement in care	−0.006	−0.000	−0.000	0.022	0.011
**Unadjusted linear network autocorrelation models**					
Individual HIV serostatus	Model a: ρ=0.077, SE 0.061; *P*=.21	Model b: ρ=0.048, SE 0.054; *P*=.38	Model c: ρ=0.065, SE 0.070; *P*=.36	Model d: ρ=−0.174, SE 0.139; *P*=.21	Model e: ρ=−0.003, SE 0.067; *P*=.97
Engagement in care	Model a: ρ=0.016, SE 0.056; *P*=.77	Model b: ρ=0.029, SE 0.052; *P*=.58	Model c: ρ=0.051, SE 0.067; *P*=.45	Model d: ρ=0.210, SE 0.136; *P*=.12	Model e: ρ=0.114, SE 0.066; *P*=.08
**Linear network autocorrelation model, adjusted for HIV serostatus**					
HIV serostatus (covariate)	Model f: *β*=−.268, SE 0.048; *P*<.001^a^	Model g: *β*=−.281, SE 0.049; *P*<.001^a^	Model h: *β*=−.231, SE 0.056; *P*<.001^a^	Model i*: β*=−.252, SE 0.079; *P*=.001^a^	Model j: *β*=−.256, SE .047; *P*<.001^a^
Engagement in care (ρ)	Model f: ρ=0.018, SE 0.054; *P*=.74	Model g: ρ=0.048, SE 0.052; *P*=.35	Model h: ρ=0.043 SE 0.065; *P*=.51	Model i: ρ=0.253, SE 0.13; *P*=.055	Model j: ρ=0.128, SE 0.064; *P*=.045^a^

a *P*<.05.

## Discussion

### Principal Findings

Sociocentric network study designs are resource heavy to conduct and primary data collection is difficult. To address this gap, we aimed to develop and test a matching algorithm consisting of multiple types of nonsociocentric networks. We generated a fuzzy matched sociocentric-like network based on the egocentric social networks, egocentric sexual networks, peer referral ties, egocentric mobile phone contact data, and Facebook network present within the N2 cohort study of Black SGM. In addition to analyzing a fuzzy match algorithm, we found that sociocentric networks and digital networks have high utility in unveiling hidden connections. More specifically, we found that in the “fuzzy” network, bridging (ie, betweenness centrality) differed based on the HIV care cascade and engagement in HIV-related care had an autocorrelative effect; these findings were not identified in the partial networks. To our knowledge, this is one of the first studies to harmonize multiple networks to construct a larger fuzzy network, to examine the peer effects of network-level, status-neutral HIV care at the individual level, and to prioritize Black SGM social networks. Our methods and findings are novel and contribute toward the fields of social and digital epidemiology by introducing a fuzzy matching algorithm that can identify hidden ties among participants within different types of networks to better understand the larger sociocentric network.

The initial peer referral chain identified 362 existing ties within our network; however, by including alternative types of networks such as social networks and sexual partner networks, phone contact data, and Facebook friends lists, we were able to identify a total of 2054 undirected ties among 400 participants. The egocentric network quintupled the network degree and doubled density while introducing an additional >50 participants into the network. Whereas the peer referral network had 19 connected components, the final and more complete network brought together these networks into one connected component. In addition, the network diameter decreased from 18 to 9. This means that, while the most distant pairs of nodes required 18 steps at maximum to reach each other, this number halved to 9 steps in the fuzzy match network. In addition, the network radius decreased from 9 to 5, meaning it did not take more than 5 steps for anyone in the network to reach someone else. The characteristic path length, or what Milgram (1967) [49] referred to as “degrees of separation,” also halved from 7.64 to 3.49 steps from the peer referral ties to the fuzzy network. Although the Chicago MSA is indeed a small world, and our population may be too homophilous for a pair of individuals to be selected at random, our findings support the idea of the “small world problem” or the probability that 2 random people selected from a larger population will know each other. Our findings support the need for study designs using peer referral to include additional estimators to account for the interdependence of participants, as these networks may not be as apparent. Further, our study supports the utility of using a fuzzy match algorithm to construct a larger sociocentric network based on other networks, which scientists may collect at a lower burden. Our findings must be interpreted with caution as the network autocorrelation parameter was small. In real-world settings, this may suggest that there is weak social influence within the fuzzy-like network, networks are diverse or mixed and have low homophily, or individual variability may be more important than network influence.

The digital data networks identified the most ties and had superior network density, degree, diameter, radius, and characteristic path length relative to the egocentric confidant and sexual networks and the peer referral network. Interestingly, the characteristic path length of the Facebook network, 2.28 steps, is lower than reported in another study, which found pairs of random people on Facebook to have a characteristic path length of 4 steps [50]. Although our study included mobile phone contact networks and Facebook networks, future studies may have better success in identifying networks using other types of digital networks based on their priority population. For example, studies prioritizing people aged 18‐29 years may have success using Instagram as 78% of people in the 18‐29 year age group use Instagram, which is higher than other age groups (59% of people aged 30‐49 years, 35% of people aged 50‐64 years, and 15% of people aged ≥65 years) [14]. If studies hope to reach Black populations, TikTok or Instagram may be the best social networking sites as 49% of US Black adults report using TikTok (relative to 28% of White adults, 49% of Hispanic adults, and 29% of Asian adults) and 46% report using Instagram (relative to 43% of White adults, 58% of Hispanic adults, and 57% of Asian adults). In addition, after the COVID-19 pandemic stay-at-home orders in the United States, social media platforms became an online “home” for many SGM [51,52]; however, further study on social media use by young (ie, 16‐34 years old) Black SGM is warranted.

Our findings suggest that, while we did not identify differences in betweenness centrality based on a participant’s HIV care cascade in the partial networks, we did so in the fuzzy-like network. More specifically, we found that participants who were virally suppressed had lower betweenness centrality measures relative to individuals in the other HIV care cascades (ie, people who did not use PrEP, people who used PrEP, and people who were not virally suppressed). This means that, relative to individuals who are HIV-negative or not virally suppressed, participants who are virally suppressed appear to be a bridge in the network less often. This could be due to their lower likelihood to engage in behaviors with enhanced vulnerability to HIV such as condomless sex [53], which could also reduce their network connections. People who are virally suppressed may also be embedded in clinical networks instead of other social, sexual, or other community networks, as they are engaged in their HIV-related care. People who are virally suppressed may not only have smaller networks but also have networks that are more closely knit, which could reduce their bridging. Alternatively, people living with HIV may also experience stigma from HIV-negative people, which could reduce their network bridging potential. Additionally, research can explore the relationship between bridging or betweenness centrality and HIV care cascade outcomes.

Our findings must be considered within their context. There is a dearth of studies comparing influence in digital and in-person networks, and it is difficult to ascertain whether endogenous influence and social norms can be transmitted through digital or in-person interactions. Additionally, the weight social media influencers and content creators may have on social norms surrounding health and how social media algorithms “push” specific content through are largely unexplored. Further, we were unable to ascertain which relationships may be more important to intervene on. Finally, within social epidemiology and social network studies, the contextual factors of the sample as well as the geographic setting are important to consider as findings may (or may not) apply to other marginalized populations or geographic settings. For example, Black SGM in Chicago, Illinois, experience intersectional discrimination [20-23] and health disinvestment within their communities [54], which negatively impact health outcomes including HIV-related outcomes. Within this population, social norms may influence access to resources, support, and other opportunities [55-58]. These findings may translate to other Black SGM born in the United States, but may not translate to Black SGM born elsewhere who migrated to the United States or Black SGM living in cities with a more robust health infrastructure. Future studies could explore these topics.

Sociocentric data, the “gold standard” of social network data, are difficult to collect due to resource demands and complex study designs. However, constructing sociocentric networks from digital data may surmount challenges such as limitations in data collection as asking participants to list everyone that they know may be subject to measurement issues like recall bias. Although our inclusion of digital data expanded the breadth of our sociocentric network, the depth, or the influence of individuals in digital networks, is unknown. In addition, although we found that the inclusion of digital data resulted in a model that found statistical significance in HIV-related behaviors, it is unclear if this model is more precise or accurately describes the data. Thus, to see if the quality or strength of digital networks may be similar to that of in-person networks, future studies identifying the quality of these ties are needed. Another way to do so could be to analyze a behavior that is more susceptible to network structure, such as direct interactions among participants (eg, likes, comments, reactions). Although our findings support the collection of additional network data, from an ethical perspective, this may increase the likelihood that a participant may be identified as part of a network that one may not want to be identified with. These concerns must be considered in study designs.

### Limitations

Network analyses are often not perceived to be generalizable because they are representative of one network at a given time point; however, we argue that the purpose of this analysis is not to generalize our findings to the larger population but rather to explore novel fuzzy matching methods to enrich social network research by identifying opportunities to harmonize multiple types of data to triangulate a more robust and complete network. Differentiating selection from peer effects in this analysis may manifest differently based on the mechanisms at play and the networks being considered—for this, Simulation Investigation for Empirical Network Analysis (SIENA) models may allow for the distinguishing between these 2 mechanisms. In addition, participants were asked to provide their perceptions of the sociodemographic information of their network members. Race and ethnicity are complicated social identities that can be incorrectly perceived by others. This could introduce misclassification as participants may incorrectly estimate their network member’s sociodemographic information and introduce bias into our findings by creating incorrect linkages between participants and creating errors in the network structure [9]. This could then trickle down and impact our tests of differences and associations and, if nonrandom, create confounding effects. Because the majority of participants felt close or very close (>80%) to their social members, we believe that misclassification bias may be minimized. A future study could also assess the accuracy of this fuzzy matching analysis. This type of data validation could include a brief survey or interviews with a random sample of the network to authenticate relationships. Another limitation is the number of participants for whom we were able to download complete Facebook data (n=144, 35%). If we were able to include Facebook data from additional participants, we could have potentially constructed a larger fuzzy network. However, because we were able to identify participants who did not provide their Facebook data from the Facebook friends lists of current participants, we believe this bias may be minimized. Future studies can opt to collect less Facebook data, such as only collecting friendship networks, instead of collecting data on group membership, comments, and other Facebook interactions. Finally, we used a cross-sectional linear network autocorrelation, a model that assumes continuous dependent variables, to analyze binary variables. The use of this model assumes that associations are linear, which may not hold for binary variables that constrain probabilities within the (0,1) range. The use of the linear network autocorrelation model may introduce misestimation of effect sizes due to nonlinearity, predictions outside the valid probability range, and heteroskedasticity. To address this bias, we have added supplemental analyses of unadjusted logistic regressions to test the effects of the network exposure to HIV serostatus and engagement in care. To establish peer effects, longitudinal data and a robust set of covariates could be used instead. Our findings may not be capturing peer effects, but rather homophily in the select effect (ie, participants deliberately seek others similar in their HIV care to be friends with). As our study was a pilot approach, our findings on autocorrelative effects may be interpreted with caution as they are marginally significant correlations.

Although not a limitation, a notable feature of this analysis was the time intensity of the match between participants and the social alters and sexual partners listed by other participants. This match of respondents to social network members and sexual network members generated over 8000 potential matches. We manually validated each match to determine if it was a true match or a false-positive match. This was a time-consuming analysis that unveiled <1% of ties of the final network. A machine learning algorithm, if trained appropriately, may alternatively be used to identify matches.

### Conclusions

Digital networks, which we operationalized as phone contact and Facebook networks, were the most productive in identifying ties and may be best to characterize social networks. Our experimental approach identified opportunities to characterize networks more thoroughly and accurately. Future studies can explore how to operationalize and examine the influence of ties across various networks. In addition, there are added benefits to including digital networks to egocentric networks as they can identify hidden ties. There are clearly differential impacts of digital data networks on health and HIV outcomes; future studies could more thoroughly assess these networks. Thus, fuzzy matching may be an effective means to identify hidden ties among participants within different types of egocentric and digital networks to better understand the larger sociocentric network, including among vulnerable populations.

## Abbreviations

ART: antiretroviral treatment
LNAM: Linear Network Autocorrelation Mode
MSA: Metropolitan Statistical Area
N2: Neighborhoods and Networks
PrEP: pre-exposure prophylaxis
QAP: Quadratic Assignment Procedure
SGM: sexually minoritized and gender expansive people
STROBE: Strengthening the Report of Observational Studies in Epidemiology
